# Biodegradation of metoprolol in oxic and anoxic hyporheic zone sediments: unexpected effects on microbial communities

**DOI:** 10.1007/s00253-021-11466-w

**Published:** 2021-08-02

**Authors:** Cyrus Rutere, Malte Posselt, Adrian Ho, Marcus A. Horn

**Affiliations:** 1 Department of Ecological Microbiology, University of Bayreuth, Bayreuth, Germany; 2 Institute of Microbiology, Leibniz University Hannover, Herrenhäuser Straße 2, Hannover, Germany; 3 Department of Environmental Science, Stockholm University, Stockholm, Sweden

**Keywords:** Metoprolol, Hyporheic zone, Micropollutant transformation, 16S rRNA microbiome analysis

## Abstract

**Abstract:**

Metoprolol is widely used as a beta-blocker and considered an emerging contaminant of environmental concern due to pseudo persistence in wastewater effluents that poses a potential ecotoxicological threat to aquatic ecosystems. Microbial removal of metoprolol in the redox-delineated hyporheic zone (HZ) was investigated using streambed sediments supplemented with 15 or 150 μM metoprolol in a laboratory microcosm incubation under oxic and anoxic conditions. Metoprolol disappeared from the aqueous phase under oxic and anoxic conditions within 65 and 72 days, respectively. Metoprolol was refed twice after initial depletion resulting in accelerated disappearance under both conditions. Metoprolol disappearance was marginal in sterile control microcosms with autoclaved sediment. Metoprolol was transformed mainly to metoprolol acid in oxic microcosms, while metoprolol acid and α-hydroxymetoprolol were formed in anoxic microcosms. Transformation products were transient and disappeared within 30 days under both conditions. Effects of metoprolol on the HZ bacterial community were evaluated using DNA- and RNA-based time-resolved amplicon Illumina MiSeq sequencing targeting the 16S rRNA gene and 16S rRNA, respectively, and were prominent on 16S rRNA rather than 16S rRNA gene level suggesting moderate metoprolol-induced activity-level changes. A positive impact of metoprolol on *Sphingomonadaceae* and *Enterobacteriaceae* under oxic and anoxic conditions, respectively, was observed. Nitrifiers were impaired by metoprolol under oxic and anoxic conditions. Collectively, our findings revealed high metoprolol biodegradation potentials in the hyporheic zone under contrasting redox conditions associated with changes in the active microbial communities, thus contributing to the attenuation of micropollutants.

**Key points:**

*• High biotic oxic and anoxic metoprolol degradation potentials in the hyporheic zone.*

*• Key metoprolol-associated taxa included Sphingomonadaceae, Enterobacteraceae, and Promicromonosporaceae.*

*• Negative impact of metoprolol on nitrifiers.*

**Supplementary Information:**

The online version contains supplementary material available at 10.1007/s00253-021-11466-w.

## Introduction

The occurrence of residual organic micropollutants such as pharmaceuticals in receiving rivers is of great concern since their long-term ecotoxicological effects remain unknown (Posselt et al. 2018). Metoprolol, a β-blocker used in the management of cardiovascular diseases, is among the top 200 drugs prescribed in the USA and Canada, while up to 100 tons are consumed in Germany annually (Scheurer et al. 2010). Due to incomplete removal in wastewater treatment plants (WWTPs), metoprolol and its metabolites can be found in the WWTP effluents and adjacent receiving rivers (Jaeger et al. 2019; Souchier et al. 2016). Potential metoprolol toxicity at μg l^-1^ to mg l^-1^ levels has been reported for all trophic levels from autotrophs (algae) to fish (Rubirola et al. 2014). Consequently, the continued discharge into the surface streams poses a potential biomagnification risk to the aquatic organisms, and a risk to humans when the surface water is used for the production of drinking water.

Microbial removal of metoprolol in the hyporheic zone of receiving rivers has recently been reported (Posselt et al. 2018; Schaper et al. 2018). However, the candidate microorganisms involved in its degradation in the hyporheic zone remain unknown. Moreover, with the hyporheic zone naturally characterized by an oxic benthic and underlying anoxic sediments (Galloway et al. 2019; Schaper et al. 2019), it is expected that the microbial metoprolol degraders and associated biodegradation potentials in these redox-delineated sediment zones vary.

Thus, the present study investigated the effect of redox conditions on metoprolol removal and associated microbial community structure in hyporheic zone sediments. Our objectives were to (i) determine metoprolol biodegradation potentials in hyporheic zone sediments under oxic and anoxic conditions, (ii) assess the response of the indigenous bacterial communities in metoprolol-impacted oxic and anoxic hyporheic zone sediments, and (iii) identify metoprolol tolerant as well as sensitive microbes.

## Materials and methods

### Chemicals

Analytical grade metoprolol tartrate used in this study was purchased from Sigma-Aldrich (Germany). Tartrate is an easily available carbon source for microorganisms; thus, tartrate-free metoprolol was prepared to avoid tartrate effects on the microbial community structure. Tartrate was separated from metoprolol by desalting using a reverse-phase C_18_ flash column (BUCHI Labortechnik AG, Switzerland). The flash column was initially activated with five column volumes methanol followed by five column volumes of water. 1.0 g of metoprolol tartrate was dissolved in 10 ml water and added to the column. The tartrate salt was washed out with three column volumes of water, and then, the metoprolol was eluted with acidified pure methanol using sulfuric acid in a step gradient (5 to 50% methanol). Metoprolol absorbance characteristics were checked at 222 nm using ultraviolet-visible (UV-VIS) spectrophotometry (Shimadzu, Japan) and at 210 nm using an Agilent Series 1200 diode array detector (DAD) (Agilent Technologies, CA, USA). Pure metoprolol was subsequently concentrated *in vacuo* via rotary evaporation of the solvent. The concentrated metoprolol was dissolved in water to make a 1 M stock solution.

### Site description and sample collection

Sediment and water samples were collected from river Erpe, an urban lowland river in Berlin, Germany, in June 2016 during a joint study within the HypoTRAIN project on the fate of trace organic compounds (TrOCs) in the hyporheic zone. The sampling site was located at Heidemühle, approximately 1 km from the municipal WWTP Münchehofe that discharges effluents into the river accounting for 60–80% of total water flow (Jaeger et al. 2019). Preliminary analysis of the redox conditions in the bulk sediment porewater at this site during the time of sampling indicated that the upper 30 cm of the hyporheic zone was oxic, with the anoxic zone occurring > 30 cm depth (Schaper et al. 2019). Therefore, sediment samples to investigate the potential degradation of metoprolol under oxic and anoxic conditions were obtained from the upper 0–10 and 30–40 cm depths, respectively. Sediment cores were collected at nine random locations along a 10-m transect using 6-cm-diameter sediment corers (Uwitec, Mondsee, Austria). The river surface water was also collected at the same transect and stored in sealed bottles. Average *in situ* metoprolol concentrations ranged from 0.4 to 5.5 μg l^-1^ in river surface and sediment pore water 2 months before sampling, and evidence for ongoing *in situ* degradation was likewise obtained (Posselt et al. 2018). The sediment cores were subsequently sliced into discrete 10-cm layers, placed in sterile Whirl-Pak sampling bags (Merck, Germany), and transferred to the laboratory in airtight coolers at 4°C for further processing. The freshly collected sediment layers were pooled per sampling depth and manually homogenized, resulting in triplicate mixed samples per depth. The sediment was stored at 4°C for no longer than 1 week prior to preparation of microcosms.

### Microcosm setup and sampling

Sediment microcosms were prepared within 1 week after sediment sampling in triplicates. Each microcosm contained 40-g sediment (fresh weight) and 120-ml river water according to OECD 308 test guidelines (Ericson 2007). The sediment microcosms were prepared in sterilized 250-ml conical flasks sealed with Steristoppers® (Heinz Herenz, Germany) for oxic incubations and in 250-ml glass bottles sealed with rubber septa bound-screw caps (Sigma-Aldrich, Germany) for the anoxic incubations. The anoxic microcosms were then purged for 1 h with pure nitrogen to remove any residual oxygen in the headspace. All microcosms were preincubated for 14 days at 20 °C in the dark to acclimatize and degrade any residual metoprolol and minimize easily degradable dissolved organic matter. Following preincubation, metoprolol was supplemented from the 1 M stock solution to the microcosms at final concentrations of 15 and 150 μM. Biotic and abiotic controls were set up in parallel. The biotic controls were microcosms not supplemented with exogenous metoprolol. The abiotic “sorption controls” consisted of autoclaved sediment and river water in the same ratio as biotic microcosms and were supplemented with 150 μM metoprolol to account for losses via sorption. The abiotic “abiotic decay controls” contained river water only. Both abiotic sets of control microcosms were incubated under anoxic conditions. Incubation was at 20 °C in the dark. Oxic microcosms were incubated with shaking at 100 rotations per minute, and anoxic microcosms were incubated under static conditions to mimic *in situ* conditions (i.e., high water flow in the upper oxic sediments and low flow conditions in the anoxic lower sediments). Slurry samples (i.e., river water-sediment suspension) for chemical analyses were withdrawn from agitated (oxic) and static (anoxic) microcosms using syringes at regular intervals during incubation. The samples were subsequently centrifuged (13,000×*g*, 5 min) and the supernatant (i.e., the aqueous phase) filtered using 0.2-μm-pore diameter regenerated cellulose membrane filters (Agilent Technologies, CA, USA). Sediment samples were likewise obtained from agitated (oxic) and static (anoxic) microcosms at days 0, 65, and 120, flash-frozen, and stored at − 80°C for subsequent extraction of nucleic acids that started within 24 h after the end of incubations.

### Analytical techniques

Metoprolol concentration was measured using high-performance liquid chromatography (HPLC) with a Zorbax SB-C18 column and an Agilent 1200 series DAD (Agilent Technologies, CA, USA). The mobile phase consisted of 0.2% trifluoroacetic acid (TFA) in deionized water (solvent A) and 0.16% TFA in acetonitrile (solvent B). Analysis was performed under gradient conditions starting with 95% A/5% B for 15 min followed by 50% A/50% B for 5 min, 100% B for 0.1 min, and finally, 15% A/85% B for 4.9 min. The flow rate was 0.5 ml min^-1^, and the column oven temperature was set at 40 °C. Metoprolol eluted after 19.6 min as indicated by an absorbance signal used for quantification at 210 nm. Concentration was determined using external standards prepared in deionized water.

Following the third feeding (second refeeding), the 15 μM metoprolol treatment was chosen as a representative setup for analysis of metoprolol transformation products. Metoprolol transformation and formation of two key transformation products, i.e., metoprolol acid (MTPA) and α-hydroxymetoprolol (α-HMTP), were assessed using a previously validated direct injection-ultra HPLC method coupled to tandem mass spectrometry (UHPLC-MS/MS) (Posselt et al. 2018). Briefly, a sample volume of 800 μl was combined with 195 μl methanol and the isotope-labeled internal standard mix in 5 μl methanol, thoroughly mixed, and then, filtered (Filtropur S 0.45 μm, PES membrane, Sarstedt, Germany) into 2-ml vials (Thermo Scientific, Germany). A quantitative and qualitative product ion was monitored for each target, and the internal-labeled standard method was used for quantification. The levels of detection and quantification were both 100 ng l^-1^ for the targets. The injection volume was 20 μl. Two blank samples to control for potential carry-over or other contamination as well as a quality control standard were injected every 15 samples. No blank contamination was observed. The precision determined with the quality control standard indicated a per cent relative standard deviation (% RSD) of 3.4, 3.8, and 11.7 for metoprolol, MTPA, and α-HMTP, respectively. Further information on chromatography, MS/MS settings, quantification, and further QA/QC data can be found in Posselt et al. (2018).

The concentration of nitrate was determined colorimetrically using a modified published protocol (Cataldo et al. 1975). To remove nitrite, 1-μl amidosulfuric acid (10%) was added to a 5-μl sample, mixed thoroughly, and incubated at room temperature for 5 min. 20 μl of 5% salicylic sulfuric acid was added and further incubated for 30 min at room temperature, followed by the addition of 167 μl 6 M NaOH. The samples were then cooled, and the developed yellow dye was measured at 410 nm using a Tecan Infinite® 200 PRO multiplex plate reader (BioTek, Germany). Concentrations of dissolved iron and manganese were relatively low (< 0.05 mgL^-1^) in the sediment at this site indicating that iron and manganese reduction were marginal as previously reported (Schaper et al. 2019). pH was determined using a portable pH meter (InLab 422; Mettler Toledo GmbH, Germany).

### Extraction of nucleic acids and reverse transcription

DNA and RNA were coextracted from the metoprolol-amended and biotic control microcosms following the published rapid protocol for coextraction of DNA and RNA from natural environments (Griffiths et al. 2000). DNA was obtained through the digestion of RNA using DNase-free RNase, while RNA was obtained by digesting DNA using RNase-free DNase (Promega, Germany), following manufacturer’s instructions. DNA and RNA concentrations were then quantified with Quant-iT® PicoGreen DNA and RiboGreen RNA assay kits (Invitrogen), respectively, using a Tecan Infinite® 200 PRO multiplex plate reader (BioTek, Germany). RNA was subsequently subjected to reverse transcription into complementary DNA (cDNA) using random hexamer primers and Superscript™ IV Reverse Transcriptase (Invitrogen, Germany) following manufacturer’s protocol.

### Amplicon generation, sequencing, and processing

Amplification and sequencing of the bacterial 16S rRNA and 16S rRNA gene amplicons were commercially performed at LGC Genomics GmbH (Berlin, Germany). Briefly, the PCR consisted of 1 × MyTaq buffer containing 1.5 units MyTaq DNA polymerase (Bioline, UK) and 2 μl of BioStabII PCR Enhancer (Sigma, Germany), 15 pmol forward primer U341F and reverse primer U806R each (Sundberg et al. 2013), and 5 ng of DNA/cDNA per sample in nuclease-free water (Thermo Fischer Scientific, Germany) in a final volume of 20 μL. For each sample, the forward and reverse primers were fused to the same 10-nt barcode sequence. The PCR was carried out using the following thermal profile: 2 min at 96 °C initial denaturation followed by 30 cycles of 96 °C for 15 s, 50 °C for 30 s, and elongation at 70 °C for 90 s. The DNA concentration of target amplicons was determined by gel electrophoresis. If needed, PCRs showing low yields were run for additional 5 cycles. About 20 ng of amplicons of each sample were pooled for up to 48 samples with different barcodes. The pooled amplicons were purified with one-volume AMPure XP beads (Agencourt) to remove primer dimers, followed by an additional purification on MinElute columns (Qiagen, Germany). About 100 ng of each purified amplicon pool DNA was used to construct Illumina sequencing libraries using the Ovation Rapid DR Multiplex System 1-96 (NuGEN, The Netherlands). Illumina libraries were pooled and size selected by preparative gel electrophoresis. Sequencing was performed on an Illumina MiSeq using V3 chemistry (Illumina, CA, USA) yielding 300 base paired-end reads.

The raw sequences were preprocessed through demultiplexing of all libraries for each sequencing lane using the Illumina bcl2fastq 2.17.1.14 software. Sequencing adapter and primers were then removed, and the forward and reverse reads combined using BBMerge 34.48. Sequences containing ambiguous bases (Ns), with homopolymer stretches of more than 8 bases or with an average Phred quality score below 33, were removed followed by alignment against the 16S Mothur-Silva SEED r119 reference alignment. Subsequently, the elimination of chimera with the uchime algorithm was performed. Taxonomical classification of the sequences was then carried out against the Silva reference classification database, and sequences from other domains of life, i.e., eukaryotes, were removed. The analyzed 16S rRNA gene and 16S rRNA sequences in samples from the oxic treatments ranged from 11,404 to 49,733 and 15,045 to 49,833, respectively. Numbers of 16S rRNA gene and 16S rRNA sequences from the anoxic treatments included in the analysis ranged from 14,006 to 49,919 and 30,089 to 49,943, respectively. OTU picking was performed using the cluster.split method by clustering at the 97% identity level followed by OTU consensus taxonomical calling with integrated taxonomical classification. Representative sequences of each OTU were then queried against a filtered version of the ribosomal database project (RDP) release 11.4 reference. A summary table with taxonomy and alignment details of each OTU representative sequence was generated. The OTU relative abundance data filtered for low-abundance OTUs was subsequently generated with QIIME 1.9.0 using rarified data based on the sample with the minimum number of sequences. The sequence data was submitted to the NCBI sequence read archive (SRA) under accession number PRJNA587738.

### Statistical analyses

Alpha diversity was evaluated based on species richness, Shannon diversity, and evenness at the OTU level using PAST v3.15 (Hammer et al. 2001). Significant differences in the indices among the samples were determined using ANOVA and post hoc Tukey test after verifying normality (Shapiro-Wilk) and homogeneity of variance (Levene’s). Between-treatment bacterial community composition variability was visualized on principal coordinate analysis plots (PCoA), and significance in the differences assessed using analysis of similarity (ANOSIM) based on Bray-Curtis dissimilarity matrix. Pattern search analysis using Pearson correlation was done with the MicrobiomeAnalyst pipeline after variance and low-abundance filtering according to default settings and relative log expression data transformation of the non-rarified OTU table (Dhariwal et al. 2017). Taxa were considered affected by metoprolol when effects were dose dependent, i.e., the abundance in amplicon libraries increased or decreased with metoprolol concentration.

## Results

### Transformation of metoprolol in oxic and anoxic hyporheic zone sediments

**Fig. 1:**
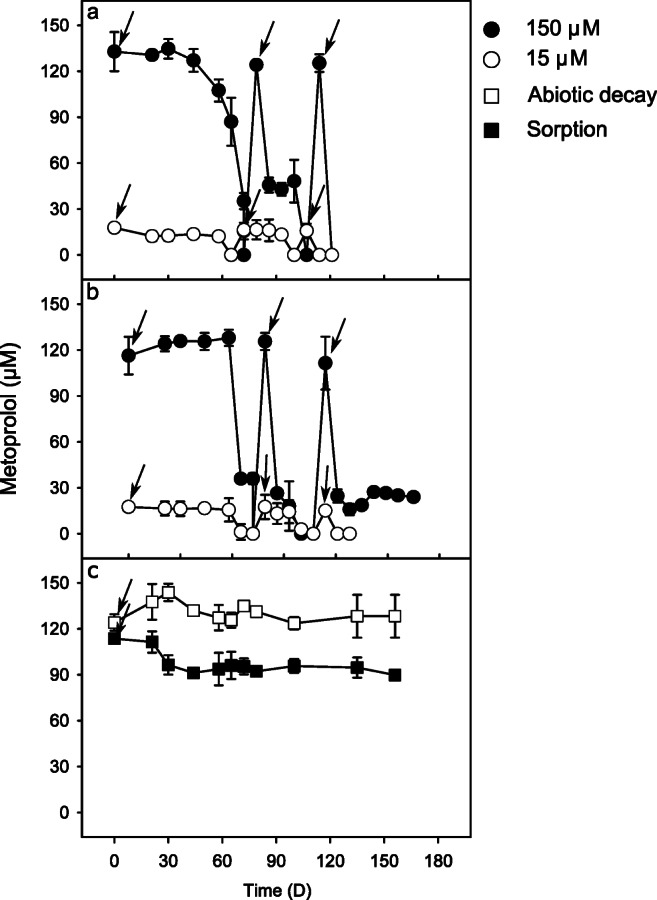
Apparent disappearance of metoprolol in the aqueous phase of hyporheic zone sediment microcosms. Biotic metoprolol removal in sediments amended with metoprolol under oxic (**a**) and anoxic (**b**) conditions, respectively. **c** Metoprolol concentrations in microcosms with autoclaved sediment and river water (“sorption”) indicative of abiotic removal of metoprolol from the aqueous phase via sorption to the sediment matrix. “Abiotic decay” indicates metoprolol concentrations in autoclaved river water microcosms without sediment to account for abiotic degradation of metoprolol. Values are the arithmetic means of triplicate incubations. Error bars represent standard deviations. Some standard deviations are smaller than the symbol size and therefore not apparent. Arrows indicate the time of refeeding of microcosms with metoprolol

The detected initial metoprolol concentration completely disappeared from the aqueous phase in the 15 and 150 μM metoprolol treatments incubated under oxic conditions within 65 and 72 days, respectively, after an initial lag phase of approximately 40 days (Fig. 1a). In the subsequent second and third refeeding, metoprolol disappearance occurred within 35 days or less, irrespective of the initial metoprolol concentration (Fig. 1a). Although the initial metoprolol concentrations measured in some of the microcosms fed with 150-μM metoprolol appeared sometimes less than the spiked concentrations, metoprolol removal was significant. Such a discrepancy might occur due to a combination of sorption and incomplete mixing. Metoprolol was always below the detection limit in non-metoprolol-amended, oxic, biotic controls (data not shown). The endogenous, initial nitrate concentrations in the oxic treatments and biotic controls declined during the first 30–60 days and marginally increased after that (Fig. S1a).

Under anoxic conditions, complete disappearance irrespective of initial metoprolol concentration occurred within 72 days following a lag phase of about 60 days (Fig. 1b). Disappearance after the second refeeding occurred within 35 days in both treatments without an appreciable lag phase. However, after the third refeeding, complete disappearance was only observed in the 15-μM treatment, while the 150-μM treatment exhibited incomplete disappearance of metoprolol (Fig. 1b). Metoprolol was always below the detection limit in non-metoprolol-amended, anoxic, biotic controls (data not shown). Nitrate concentrations decreased rapidly within the first 20 days under anoxic conditions and remained constant after that (Fig. S1b). Nitrate concentrations remained essentially constant in the sorption control (Fig. S1c).

In the abiotic controls with autoclaved sediment+river water (“sorption controls”), up to 40% of the initial metoprolol concentration disappeared from the aqueous phase, indicating sorption to the sediment matrix. Metoprolol concentrations remained essentially unchanged in the sterilized river water (abiotic decay or “hydrolysis” controls) during the entire 170-day incubation, suggesting chemical stability of metoprolol in river water under the experimental conditions (Fig. 1c).

**Fig. 2:**
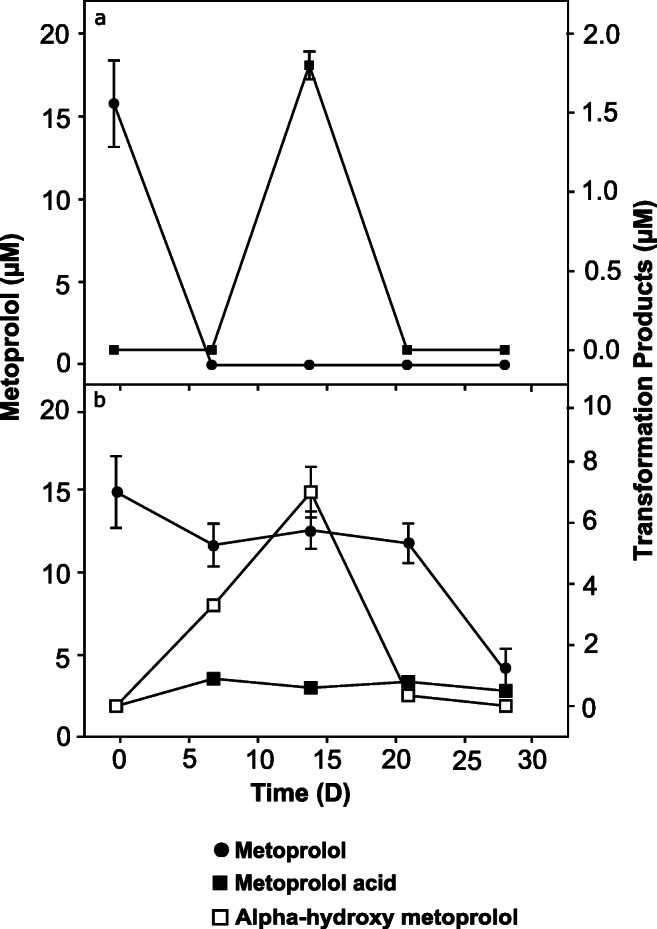
Metoprolol transformation products in hyporheic zone sediment microcosms under oxic (**a**) and anoxic conditions (**b**) subsampled from the 15-μM metoprolol treatment (following 2nd refeeding, see Fig. 1a, b). Values are the arithmetic means of triplicate incubations. Error bars represent standard deviations. Some standard deviations are smaller than the symbol size and therefore not apparent. Note different scales for the secondary axes

Transformation product analysis revealed transient formation of MTPA under oxic conditions, accounting for approximately 10% of the initial metoprolol concentration (Fig. 2a). Under anoxic conditions, MTPA and α-HMTP were formed, with α-HMTP representing the major transformation product accounting for up to 50% of the initial metoprolol concentration (Fig. 2b). Both detected transformation products subsequently disappeared in oxic and anoxic microcosms (Fig. 2). The pH was essentially constant in the microcosms during the entire incubation period and ranged from 6.4 to 6.7.

### Effect of metoprolol on bacterial diversity

**Fig. 3:**
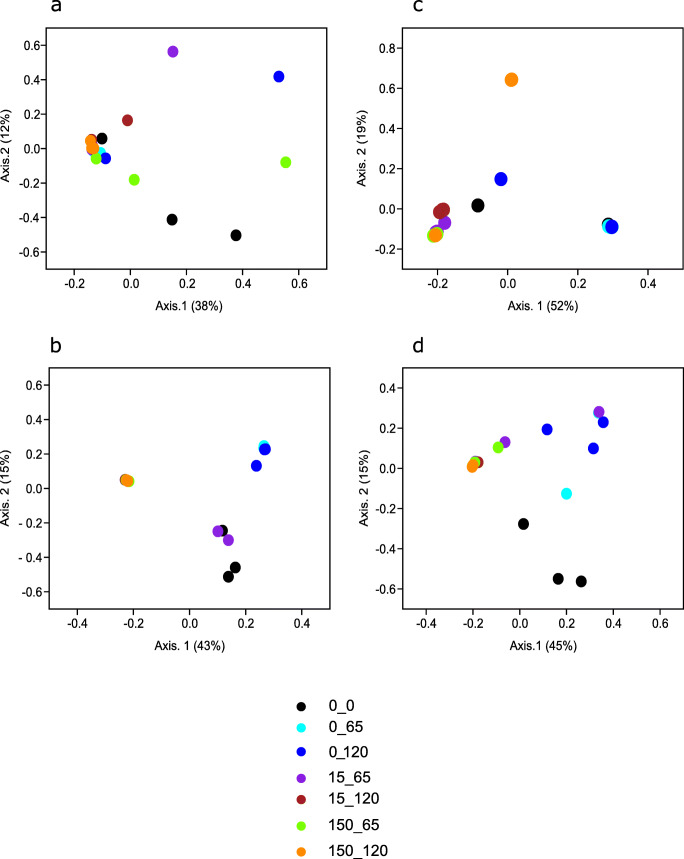
Principal coordinate analysis based on Bray-Curtis dissimilarity metric showing the effect of metoprolol treatment on the bacterial community composition on OTU level. **a**, **b** 16S rRNA gene and 16S rRNA, respectively for samples incubated under oxic conditions. **c**, **d** 16S rRNA gene and 16S rRNA, respectively, for samples incubated under anoxic conditions. Sample code: 0, 15, and 150 indicate metoprolol concentration (μM). 0, 65, and 120 at the last position in the code indicate day of sampling

16S rRNA gene and 16S rRNA-based species richness and Shannon diversity in the metoprolol-amended treatments and unamended controls in samples incubated under oxic conditions when compared to the original community indices were similar (ANOVA, *p* > 0.05; Fig. S2a, b). Likewise, no significant differences were observed in any of the 16S rRNA gene-based diversity indices under anoxic conditions, while a significant decline in the 16S rRNA level-based indices compared to the original community occurred (ANOVA, *p* < 0.05; Fig. S2d, e). The evenness followed a similar pattern as Shannon diversity (Fig. S2c, f). Clustering patterns according to metoprolol amendment but not incubation time were observed under oxic conditions on RNA level only (Fig. 3a, b; Table 1), suggesting an impact of metoprolol on the activity of certain members of the hyporheic zone microbial community under oxic conditions. Under anoxic conditions, metoprolol rather than incubation time impacted the hyporheic zone microbial community on 16S rRNA gene and 16S rRNA level (Fig. 3c, d; Table 1).

**Table 1:** Two-way ANOSIM of Bray-Curtis beta diversity with metoprolol concentration (metoprolol) and incubation time (time) as factors

Factor	Oxic^a^				Anoxic^b^			
	DNA^c^		RNA^d^		DNA		RNA	
	*R*	*p*	*R*	*p*	*R*	*p*	*R*	*p*
Metoprolol	0.04	0.200	*0.500*^e^	*0.002*^e^	*0.65*	*0.001*	*0.30*	*0.001*
Time	0.08	0.100	0.200	0.100	0.40	0.200	0.03	0.400

^a^Agitated incubation of oxic sediments

^b^Static incubation of anoxic sediments

^c^Analysis on 16S rRNA gene level

^d^Analysis on 16S rRNA level

^e^Significant effects (*p* < 0.01) are italicized

### Community composition of oxic and anoxic hyporheic zone sediments

The original oxic sediment bacterial community was composed of 12 key phyla (> 1% relative abundance) including *Proteobacteria*, *Bacteroidetes*, *Acidobacteria*, *Gemmatimonadetes*, *Nitrospirae*, *Chlorobi*, *Latescibacteria*, and *Firmicutes* based on 16S rRNA gene sequence analysis (Fig. S3a). *Proteobacteria* and *Bacteroidetes* dominated and accounted for up to 99% of the total original community composition based on 16S rRNA sequences in the oxic sediments (Fig. S3b). The dominant families (> 3% relative abundance) at both 16S rRNA gene and 16S rRNA levels included the proteobacterial families *Moraxellaceae*, *Chromatiaceae*, and *Commamonadaceae*, as well as *Bacteroidetes*-affiliated *Flavobacteriaceae* (Fig. S4a, b). In contrast, the original anoxic sediment bacterial community was dominated by *Proteobacteria* and *Chloroflexi* (Fig. S3c, d), with *Enterobacteriaceae* as the dominant family (Fig. S4c, d).

### Effect of metoprolol on microbial community structure

**Fig. 4:**
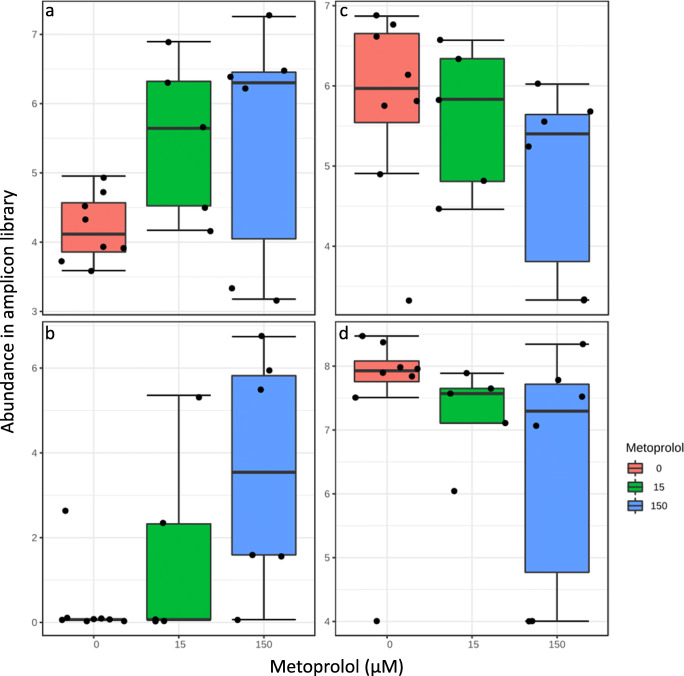
Log-transformed abundance of *Sphingomonadaceae* (**a**), *Novosphingobium* sp. (**b**), *Nitrospinaceae* (**c**), and *Nitrospiraceae* (d) affiliated, 16S rRNA-derived sequences during oxic incubations of hyporheic zone sediments

**Fig. 5:**
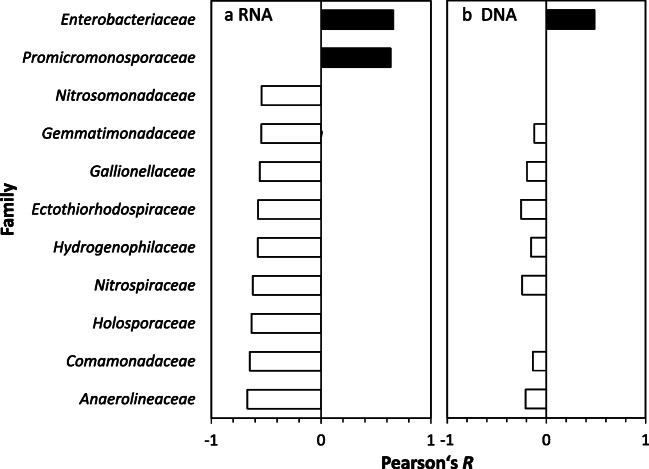
Pearson correlation of family-level taxa with metoprolol concentration during anoxic incubations on RNA (**a**) and DNA (**b**) level. Correlations on RNA level were significant (adjusted *p* < 0.1), while correlations on DNA level represent tendencies

**Fig. 6:**
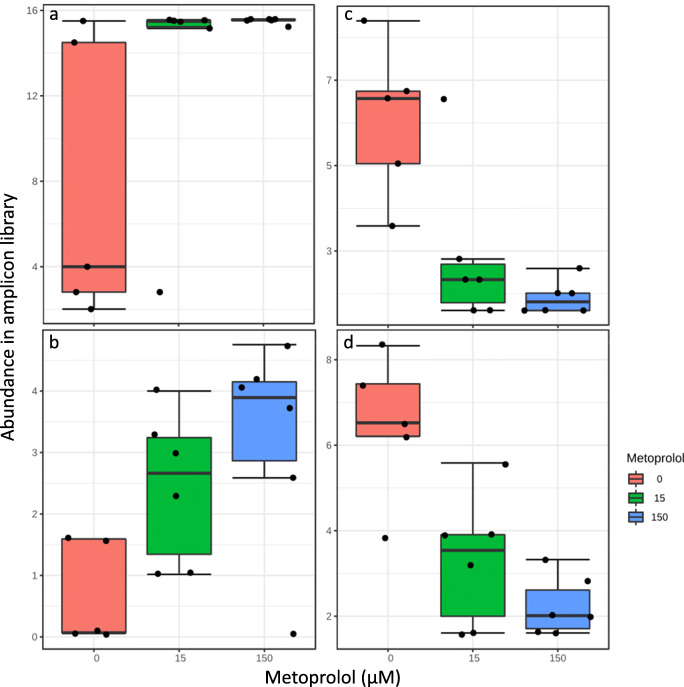
Log-transformed abundance of *Enterobacteriaceae*, i.e., *Escherichia/Shigella sp.* (**a**), *Promicromonosporaceae*, i.e., *Cellulosimicrobium* sp. (**b**), *Nitrosomonadaceae* (**c**), and *Nitrospiraceae* (**d**) affiliated, 16S rRNA-derived sequences during anoxic incubations of hyporheic zone sediments

Correlations of phyla with metoprolol were not significant under oxic and anoxic conditions on 16S rRNA gene level (Pearson’s *R*; adjusted *p* > 0.1). However, *Sphingomonadaceae*, i.e., *Novosphingobium* sp., affiliated taxa tended to increase in response to metoprolol, while *Nitrospinaceae* and *Nitrospiraceae* tended to decrease under oxic conditions on RNA level (Fig. 4). In contrast, *Proteobacteria* increased significantly in relative abundance in metoprolol treatments under anoxic conditions at all time points relative to unamended controls on 16S rRNA level (Pearson’s *R* = 0.66, adjusted *p* < 0.1), while *Nitrospirae* were negatively correlated with metoprolol (Pearson’s *R* = − 0.48; adjusted *p* < 0.1; Fig. S3d). *Enterobacteriaceae* and *Promicromonosporaceae* were positively correlated with metoprolol, while many other families were negatively impacted by metoprolol, including *Nitrosomonadaceae*, *Nitrospiraceae*, and *Anaerolineaceae* (Figs. 5a and 6). Effects on DNA level showed a similar tendency for most of the taxa responding on RNA level (Fig. 5b).

## Discussion

### Microbial degradation as an important removal mechanism of metoprolol in hyporheic zone sediments

The disappearance of metoprolol was primarily attributed to biodegradation and secondarily to sorption (Fig. 1). Transient accumulation of transformation intermediates and their subsequent disappearance allow for a complete removal of metoprolol (Fig. 2). Our findings are consistent with previous studies conducted *in situ* on the same river that reported both biodegradation and sorption as the critical attenuation mechanisms for metoprolol in the hyporheic zone (Posselt et al. 2018; Schaper et al. 2019). Similar findings were reported in water-sediment recirculating flumes and sediment microcosms (Mechelke et al. 2020; Posselt et al. 2020; Rutere et al. 2020a). The significant contribution of biodegradation in the removal of metoprolol has further been demonstrated in other diverse matrices such as riverbank filtration systems, WWTPs, subsurface flow constructed wetlands, biological activated carbon systems, and surface water (Abromaitis et al. 2016; Li and McLachlan 2019; Rubirola et al. 2014; Rühmland et al. 2015; Schmidt et al. 2007). The contribution of sorption to the overall removal of metoprolol from the aqueous phase may vary due to factors influencing its interaction with sediments, such as pH and organic matter content (Schaper et al. 2019). In this study, the observed sorption of metoprolol to the sediment was likely influenced by the ambient pH of 6.6 measured in the microcosms. Metoprolol occurs in the cationic form at pH 6.5/6.6 as a result of the protonation of the amino moiety of the side chain (Ramil et al. 2010). Therefore, sorption to the sediment was likely due to electrostatic interactions with the sediment particles as previously reported (Schaper et al. 2019). The concentration of metoprolol remained relatively constant throughout the incubation in the river water-only incubations serving as “abiotic decay” control for determining abiotic degradation, suggesting abiotic degradation was neglectable for the attenuation of metoprolol in the dark, which is in line with the well-known chemical stability of metoprolol under pH neutral conditions (Borkar et al. 2012).

The extended lag phase before a notable metoprolol depletion occurred following initial feeding compared to the immediate onset of degradation in subsequent refeeding (Fig. 1a, b) suggests the expression of enzymes involved in its degradation and/or the enrichment of metoprolol degraders. Thus, microorganisms capable of degrading metoprolol were present *in situ* in the hyporheic zone sediments and prone to respond to metoprolol given enough time for activation. Indeed, the short hydraulic retention time (6–18 h) characteristic of most modern WWTPs (Maurer et al. 2007) is considered a contributing factor to their low efficiency in removing most emerging (micro-)pollutants (Peralta-Maraver et al. 2018). Our findings, therefore, demonstrate that longer residence times in the sediment matrix may result in enhanced degradation of compounds otherwise considered little or non-biodegradable in conventional WWTPs.

In addition to the residence time, the underlying redox conditions influence the ecological functioning of the hyporheic zone. In a comparable riverbank filtration system, redox conditions were shown to have a more pronounced effect on the removal of micropollutants such as metoprolol than residence times of the water (Schmidt et al. 2007). However, metoprolol removal rates were similar under oxic and anoxic conditions in our experiment (Fig. 1a, b). The transformation of metoprolol under anoxic conditions started when nitrate had declined to a constant level, suggesting that nitrate independent degradation pathways were active. Incomplete metoprolol disappearance under anoxic conditions was observed in the 150 μM treatment after the third refeeding (Fig. 1). Although accumulation of inhibitory metoprolol transformation products cannot be excluded, the demonstrated capabilities of the microbial community to transform major transformation intermediates (Fig. 2) suggests that depletion of nutrients or trace elements during the incubation in a closed system is also a likely explanation. The complete removal of metoprolol in the 15-μM treatment even after the third refeeding suggests the anticipated environmental relevant concentrations are likewise amenable to complete removal. Our findings are in line with a riverbank filtration system where metoprolol removal of over 80% occurred under oxic, suboxic, and anoxic conditions (Schmidt et al. 2007) and highlight the potential for complete metoprolol removal under the dynamic redox conditions characteristic of the hyporheic zone.

### Dissimilar metoprolol biotransformation pathways under oxic and anoxic conditions

Detected metoprolol transformation intermediates under oxic and anoxic conditions (Fig. 2) might be due to dissimilar degradation pathways, given that α-HMTP production outpaced α-HMTP conversion. Indeed, MTPA was detected at similar concentrations in oxic and anoxic incubations, while α-HMTP transiently accumulated to levels of initial metoprolol under anoxic conditions only. Such findings allow for the utilization of different metabolic pathways by the different bacterial communities inhabiting the redox-delineated segments of the hyporheic zone. Under oxic conditions, MTPA was detected only upon depletion of metoprolol (Fig. 2a). MTPA formation via the o-desmethylmetoprolol (O-DMTP) intermediate as previously reported in open-water wetland microcosms or via direct oxidative demethylation is a possible pathway (Fig. S5; Kern et al. 2010; Posselt et al. 2020; Svan et al. 2016). In a previous study using batch experiments with activated sludge, MTPA, α-HMTP, and O-DMTP were identified as significant metoprolol transformation products accounting for less than 5% of the initial metoprolol concentration (Rubirola et al. 2014). However, the apparent absence of α-HMTP and O-DMTP in the oxic microcosms suggests formation of MTPA directly from metoprolol via, e.g., CYP450-mediated demethylation during aerobic microbial biotransformation in the current study (Fig. S5).

Accumulation of MTPA concomitant to metoprolol degradation under anoxic conditions (Fig. 2b) indicated anaerobic O-demethylation. O-demethylation is the initial step in the biodegradation of most methoxylated aromatic compounds by anaerobic bacteria including *Escherichia* sp. of the family *Enterobacteriaceae* (Grbić-Galić 1986; DeWeerd et al. 1988; Liu and Suflita 1993). Moreover, the formation of α-HMTP likely occurred via the oxygen-independent hydroxylase activity, an ubiquitous mechanism for attacking recalcitrant substrates in the absence of oxygen, where water is the source of the hydroxyl group (Heider et al. 2016; Rabus et al. 2016). The disappearance of the transformation products (Fig. 2) under both conditions points to further biotransformation via the intermediates shown (Fig. S5) as predicted using the EAWAG-BBD Pathway Prediction System (http://eawag-bbd.ethz.ch/predict/aboutPPS.html). Taken together, it is evident that redox-delineations promote variable biodegradation pathways probably associated with different bacterial taxa occupying specific niches in the hyporheic zone.

### Moderate impact of metoprolol on key taxa of the hyporheic zone

Oxic and anoxic bacterial communities in hyporheic zone sediments supported metoprolol degradation (Fig. 1). However, effects on the microbial community structure under oxic conditions were evident for RNA rather than DNA level analyses (Table 1), suggesting that metoprolol impacted the activity of microbes but not their growth. A total of app. 450 μM of metoprolol were consumed over the incubation period, which should suffice to stimulate growth and not only activity of potential degraders as was shown for ibuprofen degrading alpha- and gammaproteobacterial strains isolated from the same hyporheic zone (Rutere et al. 2020b). However, other edaphic factors, co-metabolic degradation and/or many organisms consuming metoprolol might have resulted in limited detectable growth of metoprolol degraders. Nevertheless, *Sphingomonadaceae* (*Novosphingobium* sp.) tended to respond to metoprolol (Pearson’s *R* = 0.52, adjusted *p* > 0.1; Fig. 4a, b) and thus are considered to be associated with metoprolol degradation under oxic conditions. Indeed, *Sphingomonadaceae*-affiliated genera are widely reported xenobiotic degraders, in particular for aromatic compounds, and are associated with pollutant degradation in diverse environmental matrices including the hyporheic zone (Dallinger and Horn 2014; Liu et al. 2011a,[Bibr CR23][Bibr CR22]; Coll et al. 2020; Posselt et al. 2020; Rutere et al. 2020b).

The capability to perform O-demethylation reactions was recently discovered for *Novosphingobium aromaticivorans*, supporting the view that *Sphingomonadaceae* were associated with aerobic metoprolol degradation in hyporheic zone sediments (Perez et al. 2021). Metabolite analysis allowed for CYTP450 catalyzed O-demethylation reactions (Figs. 2 and S5). Such enzymes are widely distributed among *Streptomyces* sp. that showed a tendency to be correlated with metoprolol concentration (Pearson’s *R* = 0.50, adjusted *p* > 0.1; Kelly and Kelly 2013). Co-metabolic degradation would allow for a higher metoprolol tolerance than other taxa rather than growth. Thus, *Streptomyces* sp. associated co-metabolic degradation of metoprolol cannot be excluded under oxic conditions. In contrast, *Nitrospinaceae* and *Nitrospiraceae* accommodate well recognized nitrite oxidizers (Lücker and Daims 2014; Daims 2014) and were negatively affected by metoprolol (Fig. 4c, d), suggesting a potential of metoprolol to impair nitrite oxidation and thus nitrification activities in oxic hyporheic zone sediments.

In anoxic hyporheic zone sediments, the predominance of *Proteobacteria*-affiliated taxa in the metoprolol-amended samples indicates a significant contribution of this phylum to metoprolol removal under anoxic conditions (Fig. S4c, d). Among these taxa were *Enterobacteriaceae* (*Escherichia*-*Shigella* affiliating) that significantly correlated with supplemental metoprolol concentration on RNA level (Figs. 5a and 6a). Similar trends for *Enterobacteriaceae* were observed on DNA level, suggesting a stimulation of this family by metoprolol (Fig. 5b). *Enterobacteriaceae* including the well-studied *Escherichia coli* have the often overlooked capability to use diverse aromatic compounds as sole carbon and energy source and to perform anaerobic O-demethylation reactions that were observed during our study (Figs. 2 and S5; Grbić-Galić 1986; Diaz et al. 2001). Actinobacterial *Promicromonosporaceae* (*Cellulosimicrobium* sp.) likewise significantly correlated with metoprolol concentration on RNA level, suggesting a stimulation of its activity by metoprolol (Figs. 5a and 6b). A recently isolated *Cellulosimicrobium* sp. showed indeed polycyclic aromatic compound degradation activities under anoxic, nitrate-reducing conditions (Qin et al. 2018). Thus, *Cellulosimicrobium* along with *Enterobacteriaceae*-related taxa are likely candidates for being associated with anaerobic metoprolol degradation in the hyporheic zone. Interestingly, more taxa were impaired by metoprolol than stimulated in anoxic hyporheic zone sediments (Figs. 5 and 6c, d). *Anaerolineaceae* of the phylum Chloroflexi thrive in anaerobic digestors and waste water treatment plants and have a fermentative metabolism under anoxic conditions (Sengupta and Pal 2021). Such organisms are trophically associated with methanogens, suggesting that metoprolol might have negative consequences for the anaerobic food chain and thus the mineralization of biopolymers in anoxic hyporheic zone sediments in the absence of alternative electron acceptors other than carbon dioxide. *Nitrosomonadaceae* and *Nitrospiraceae* associated with ammonia and nitrite oxidation, respectively, declined in response to metoprolol on RNA level (Fig. 6c, d), again suggesting an impairment of nitrifying communities by metoprolol as was observed in oxic hyporheic zone sediments (Prosser 2007). Changes on DNA level in response to metoprolol were not as prominent as on RNA level (Fig. 5b), suggesting effects of metoprolol on microbial activity rather than growth.

In conclusion, the hyporheic zone bacterial community is prone to respond upon contact with metoprolol. Metoprolol was a potential inhibitor of N-cycling and anaerobic food chain associated organisms, thus highlighting the importance of its removal. Indeed, the major removal routes of metoprolol in the oxic and anoxic segments of the hyporheic zone were via biotic aerobic and anaerobic degradation mechanisms. Microbe-catalyzed metoprolol removal in the hyporheic zone under oxic and anoxic conditions is possible when hydraulic retention times in the sediment matrix are significantly higher than those in WWTPs, emphasizing the importance of contact time between diverse bacterial groups and pseudo-persistent environmental pollutants. Increasing sludge age, i.e., the mean cell residence time, during waste water treatment in WWTPs might thus be an option for increasing the removal efficiency for some of the easier to degrade emerging organic pollutants. Long half-lives of emerging organic pollutants like metoprolol will nevertheless complicate economical and complete removal in WWTPs, thus demanding for degradation in the environment. Although specific marker genes for the metoprolol degradation pathways are yet undefined, and concentrations of micropollutants might not suffice to stimulate significant growth of degraders, the use of differential abundance analysis based on 16S rRNA along with sensitive LC methods demonstrates that the hyporheic zone is a reservoir of metoprolol degrading taxa and provides a basis for understanding effects of metoprolol on microbial communities in the environment.

## Supplementary information

ESM 1 (PDF 986 kb)

## Data Availability

The datasets generated during the current study are available from the corresponding author on reasonable request. Sequence data are available at the NCBI sequence reads archive (SRA) under accession number PRJNA587738.
