# Supplementary material for: Biodegradation of metoprolol in oxic and anoxic hyporheic zone sediments: unexpected effects on microbial communities
Source: Appl Microbiol Biotechnol. 2021 Aug 2;105(14-15):6103–15. doi: 10.1007/s00253-021-11466-w (PMC8390428; doi:10.1007/s00253-021-11466-w)
Supplement: Supplementary file 1 — (PDF 986 kb) [file 253_2021_11466_MOESM1_ESM.pdf]

Supplementary Materials for ***Applied Microbiology and Biotechnology***

Biodegradation of metoprolol in oxic and anoxic hyporheic zone sediments: Unexpected effects on microbial communities

Cyrus Rutere[1,2], Malte Posselt[3], Adrian Ho[2], and Marcus A. Horn[1,2 *]

[1] Department of Ecological Microbiology, University of Bayreuth, Bayreuth, Germany

[2] Institute of Microbiology, Leibniz University Hannover, Hannover, Germany

[3] Department of Environmental Science, Stockholm University, Stockholm, Sweden

---

Running title: Metoprolol biodegradation in hyporheic zones

Keywords: Metoprolol, hyporheic zone, amplicon Illumina sequencing, micropollutant

---

* **Correspondence:** Marcus A. Horn, Institute of Microbiology, Leibniz University Hannover, Herrenhäuserstraße. 2, Germany. Tel: (+49) (0) 511-76217980. E-Mail: horn@ifmb.uni-hannover.de

Legends – Supplemental Figures

Fig. S1. Nitrate (NO_3^-) concentration during incubation in metoprolol-amended (15 µM and 150 µM) and unamended sediment microcosms under oxic (**a**) and anoxic (**b**) conditions. Panel **c** represents NO_3^- concentration in the abiotic (sorption) control amended with 150 µM metoprolol under anoxic conditions. Values are the arithmetic means of triplicate incubations. Error bars represent standard deviations. Some standard deviations are smaller than the symbol size and therefore not apparent. Please note that nitrate disappeared to "background concentrations" reflecting the detection limit of the assay, i.e. smaller than 0.05 mM. "Background concentrations" of nitrate were due to absorption of humic acids in the aqueous extract of the hyporheic zone sediments. Nevertheless, the difference in nitrate concentration was unaffected.

Fig.S2. Alpha diversity indices based on 16S rRNA gene and 16S rRNA sequences for incubations under oxic (**a – c**) and anoxic (**d – f**) conditions. Values are the arithmetic means of triplicate incubations. Error bars represent standard deviations. Some standard deviations are smaller than the symbol size and therefore not apparent. Sample code: 0, 15 and 150 indicate metoprolol concentration (µM). 0, 65, and 120 in the last position in the code indicate the day of sampling.

Fig.S3. Mean relative abundance of major bacterial phyla (> 1% relative abundance). Panels **a** and **b** correspond to 16S rRNA gene and 16S rRNA, respectively for samples incubated under oxic conditions. Panels **c** and **d** correspond to 16S rRNA gene and 16S rRNA, respectively, for samples incubated under anoxic conditions. Phyla accounting for less than 1% of all sequences are grouped as ''others''. Sample code: 0, 15 and 150 indicate metoprolol concentration (µM). 0, 65, and 120 at the last position in the code indicate day of sampling.

Fig.S4. Mean relative abundance of major bacterial families (> 3 % relative abundance). Panels **a** and **b** correspond to 16S rRNA gene and 16S rRNA, respectively for samples incubated under oxic conditions. Panels **c** and **d** correspond to 16S rRNA gene and 16S rRNA, respectively, for samples incubated under anoxic conditions. Sample code: 0, 15 and 150 indicate metoprolol concentration (μM). 0, 65, and 120 at the last position in the code indicate day of sampling.

Fig.S5. Hypothetical metoprolol biotransformation pathways in hyporheic zone sediments under oxic (**a**) and anoxic (**b**) conditions based on transformation products identified in this study and other predicted intermediates using the EAWAG-BBD Pathway Prediction System.

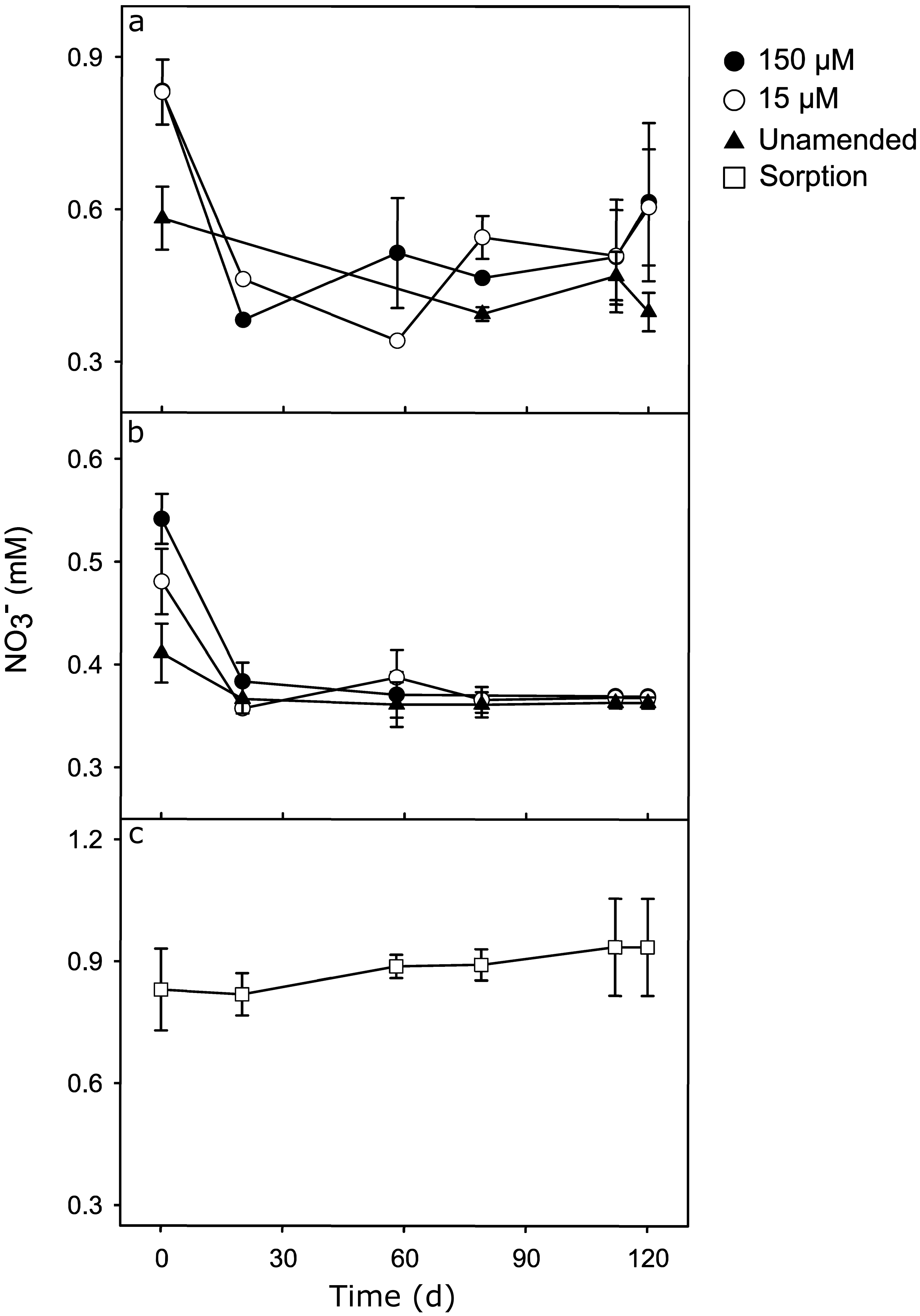

a
b
c
150 μM
15 μM
Unamended
Sorption
0.9
0.6
0.3
0.6
0.5
0.4
0.3
1.2
0.9
0.6
0.3
0
30
60
90
120
NO_3^- (mM)
Time (d)

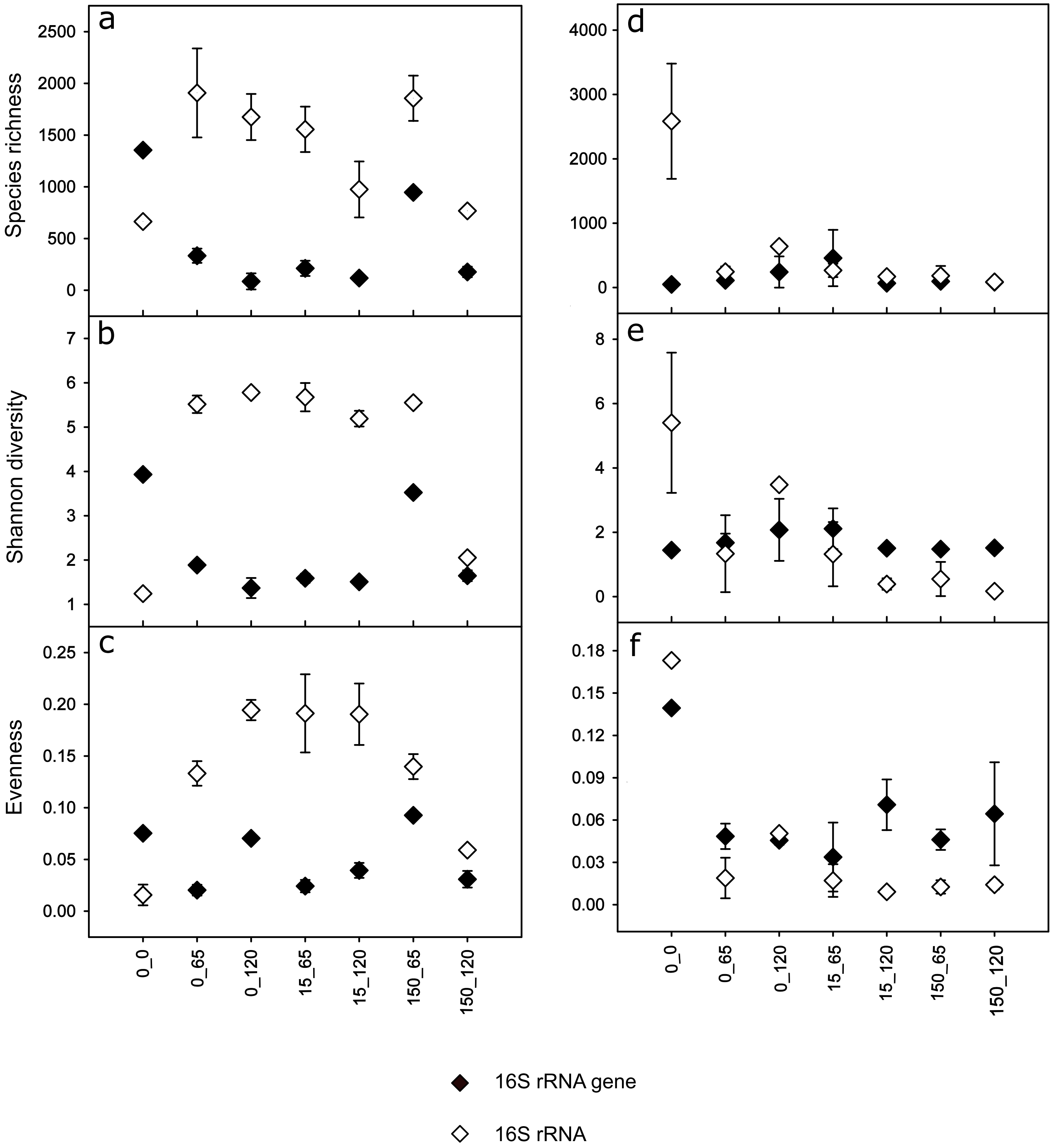

a
b
c
d
e
f
Species richness
Shannon diversity
Evenness
0_0
0_65
0_120
15_65
15_120
150_65
150_120
16S rRNA gene
16S rRNA

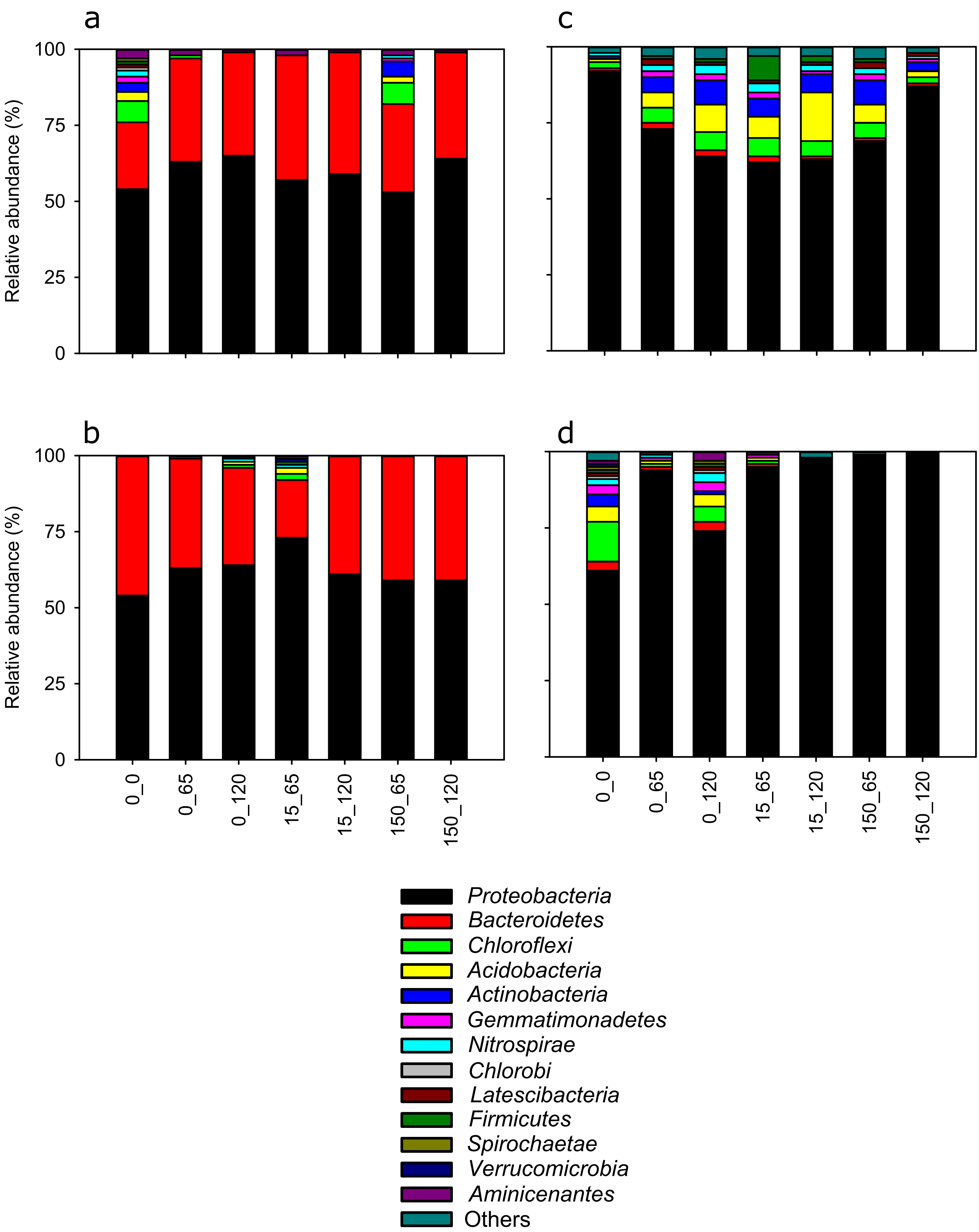

a
b
c
d
Relative abundance (%)
0
25
50
75
100
0_0
0_65
0_120
15_65
15_120
150_65
150_120
Proteobacteria
Bacteroidetes
Chloroflexi
Acidobacteria
Actinobacteria
Gemmatimonadetes
Nitrospirae
Chlorobi
Latescibacteria
Firmicutes
Spirochaetae
Verrucomicrobia
Aminicenantes
Others

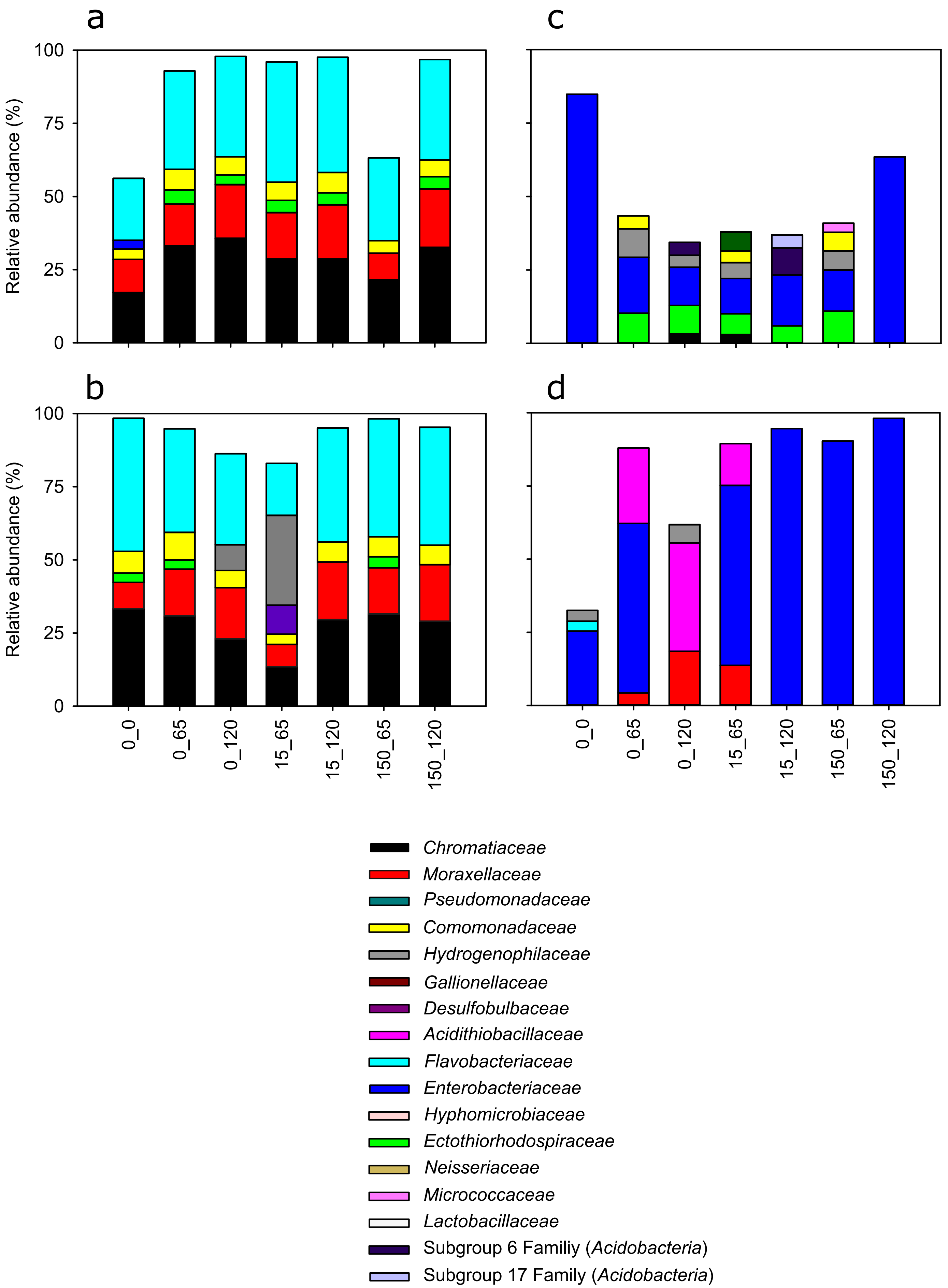
a
b
c
d
Relative abundance (%)
Relative abundance (%)
100
75
50
25
0
0_0
0_65
0_120
15_65
15_120
150_65
150_120
Chromatiaceae
Moraxellaceae
Pseudomonadaceae
Comomonadaceae
Hydrogenophilaceae
Gallionellaceae
Desulfobulbaceae
Acidithiobacillaceae
Flavobacteriaceae
Enterobacteriaceae
Hyphomicrobiaceae
Ectothiorhodospiraceae
Neisseriaceae
Micrococcaceae
Lactobacillaceae
Subgroup 6 Familiy (Acidobacteria)
Subgroup 17 Family (Acidobacteria)

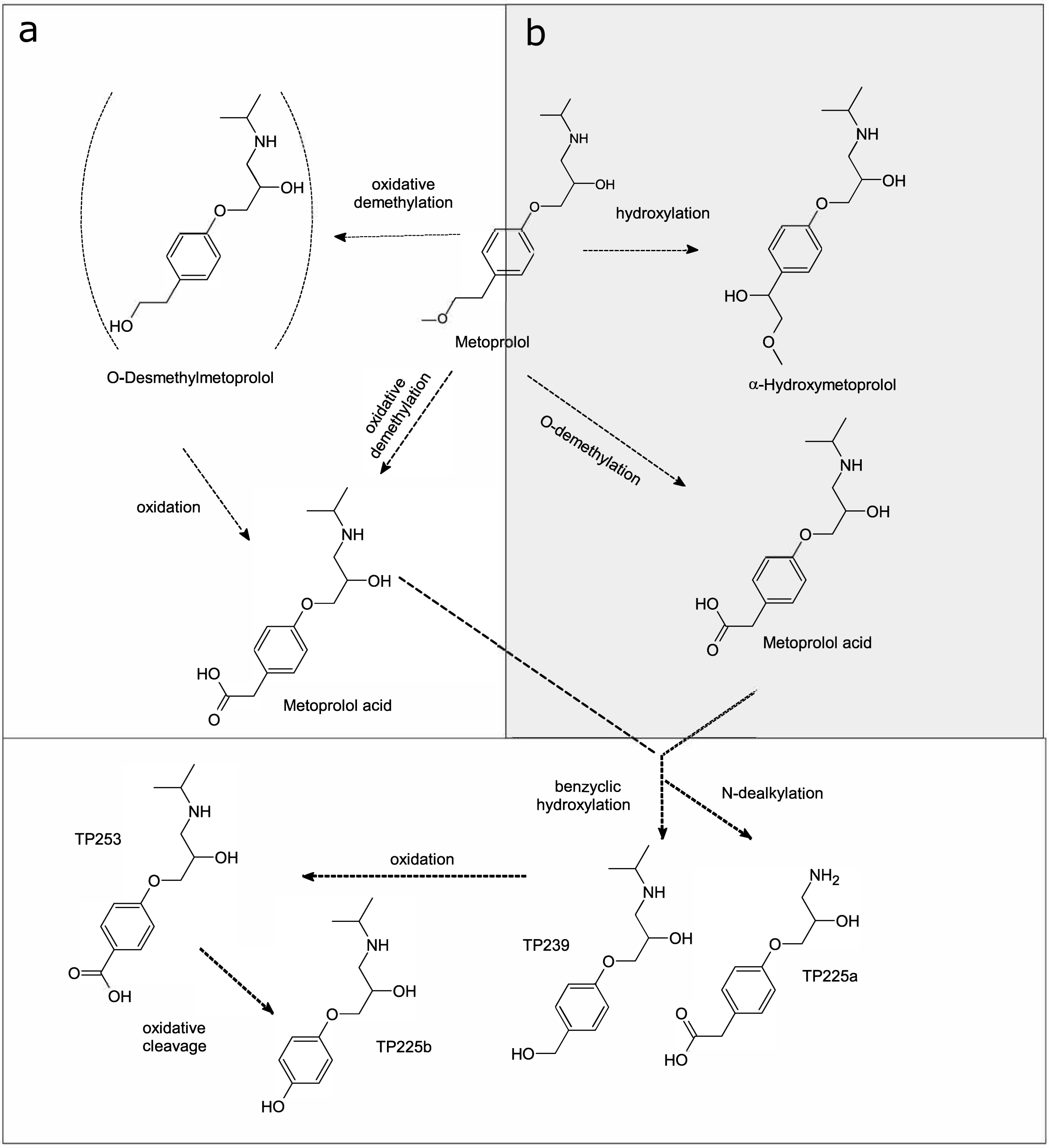

a
b
oxidative demethylation
hydroxylation
Metoprolol
O-Desmethylmetoprolol
α-Hydroxymetoprolol
oxidative demethylation
O-demethylation
oxidation
Metoprolol acid
Metoprolol acid
benzyclic hydroxylation
N-dealkylation
TP253
oxidation
TP239
TP225a
oxidative cleavage
TP225b
